# Supplementary material for: Germinal center output is sustained by HELLS-dependent DNA-methylation-maintenance in B cells
Source: Nat Commun. 2023 Sep 14;14:5695. doi: 10.1038/s41467-023-41317-3 (PMC10502085; doi:10.1038/s41467-023-41317-3)
Supplement: Supplementary file 1 — Supplementary Information [file 41467_2023_41317_MOESM1_ESM.pdf]

Supplementary Information

Germinal center output is sustained by HELLS-dependent DNA-methylation-maintenance in B cells

Clara Cousu[1]*, Eléonore Mulot[1]*, Annie De Smet[1], Sara Formichetti[2,3], Damiana Lecoeuche[1], Jianke Ren[4,5], Kathrin Muegge[4], Matthieu Boulard[2], Jean-Claude Weill[1], Claude-Agnès Reynaud[1] and Sébastien Storck[1]

Affiliations:

1. Université Paris Cité, CNRS, INSERM, Institut Necker Enfants Malades, F-75015 Paris, France
2. Epigenetics and Neurobiology Unit, European Molecular Biology Laboratory (EMBL), 00015 Monterotondo, Italy
3. Joint PhD degree program, European Molecular Biology Laboratory and Faculty of Biosciences, Heidelberg University, Germany
4. Epigenetics Section, Frederick National Laboratory for Cancer Research in the Mouse Cancer Genetics Program, National Cancer Institute, Frederick, MD, USA
5. Present address : NHC Key Lab of Reproduction Regulation,Shanghai Engineering Research Center of Reproductive Health Drug and Devices, Shanghai Institute for Biomedical and Pharmaceutical Technologies, Shanghai 200237, China

* These authors contributed equally to this paper
Corresponding author: sebastien.storck@inserm.fr

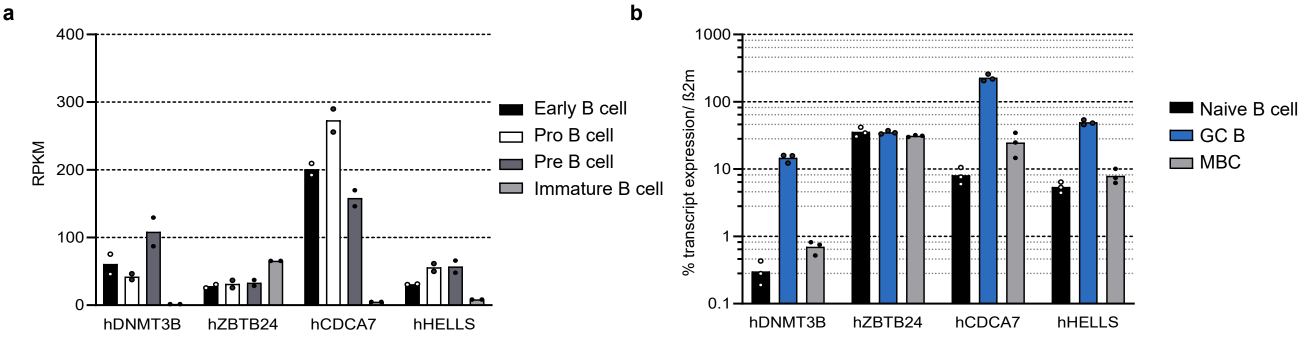

Supplementary Figure 1 – *Hells* and *Cdca7* are upregulated in human and mouse GC B cells.

(a) Expression of human *DNMT3B*, *ZBTB24*, *CDCA7* and *HELLS* in bone marrow B-cell progenitors and immature B cells (GSE115656). Each dot represents one sample (*n*=2). RPKM = reads per kb per million mapped reads.

(b) Expression of the 4 ICF genes in tonsillar naïve, germinal center (GC) and memory (MBC) from 3 independent donors (GSE110669). Bar charts and error bars represent the mean percentage of transcript expression ±SD of 3 samples. Data are normalized to β2m expression.

Source data are provided in Source Data File.

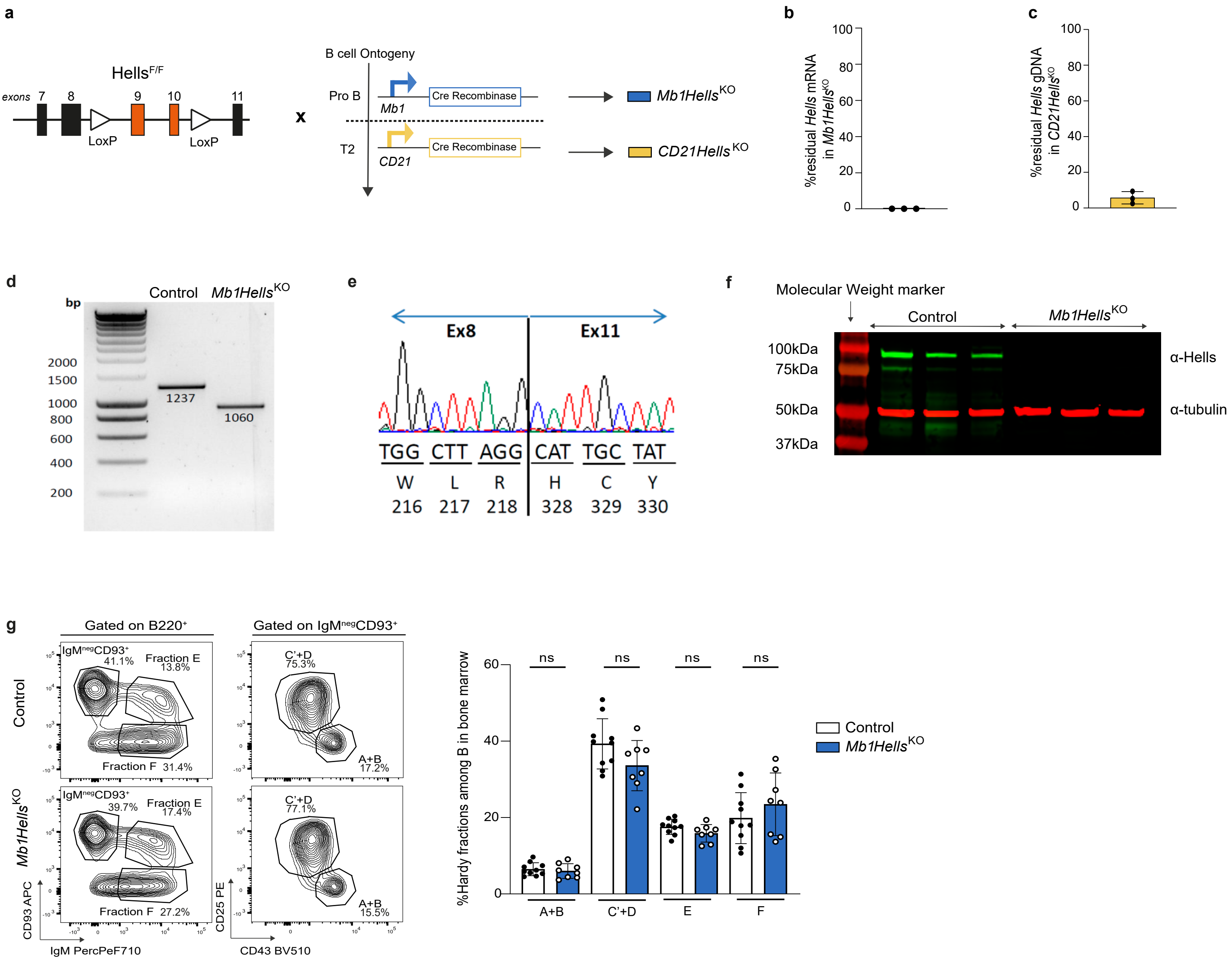

Supplementary Figure 2 – *Hells* B-cell conditional knockout mice display normal B-cell development.

(a) A floxed strain (*Hells*$^{F/F}$) with LoxP sites that flank exons 9 and 10 of Hells was crossed with *Mb1-Cre* or *CD21-Cre* mice to delete these exons in pro-B (*Mb1Hells*^{KO}) or transitional T2 B cells (*CD21Hells*^{KO}).

(b) Quantification by RT-qPCR of the residual expression of *Hells* exons 9 and 10 in sorted FoB cells (B220^{+}CD21^{mid}CD23^{mid}) from *Mb1Hells*^{KO} compared to control mice, after normalization to *Gapdh*. The bar charts and bar errors represent the mean ±SD amount of *Hells* mRNA levels. (*n*=3 mice for each genotype).

(c) Quantification of the deletion of exons 9 and 10 in *CD21Hells*^{KO} by qPCR on genomic DNA extracted from splenic non-GC B cells (B220^{+}CD95^{neg}GL7^{neg}), after normalization to *Rev3* exon 13 reference gene. The bar charts and bar errors represent the mean ±SD amount of *Hells* exons (n=3 mice for each genotype).

(d) Gel electrophoresis of retrotranscribed mRNA for one control and one *Mb1Hells*^{KO} mice.

(e) Representative electrophoregram using Sanger sequencing showing truncated cDNA amplified on *in vitro* activated B cells from one *Mb1Hells*^{KO} mice, with translated codons and positions below.

(f) Western blot analysis revealing HELLS and α-tubulin expressed in *in vitro* activated B cells from (*n*=3) control and *Mb1Hells*^{KO} mice.

(g) Representative flow cytometry plots of Hardy's fractions of B cell progenitors in the bone marrow of a control and a *Mb1Hells*^{KO} mouse. Fr.A+B: B220^{+}IgM^{neg}CD93^{+}CD43^{+}CD25^{neg}; Fr.C'+D : B220^{+}IgM^{neg}CD93^{+}CD43^{neg}CD25^{+}; Fr.E: B220^{+}CD93^{+}IgM^{+} ; Fr.F B220^{hi}CD93^{neg}IgM^{+}. Bar charts on the left show quantification of the subsets in *n*=8 *Mb1Hells*^{KO} and *n*=10 control animals, (of which 7 were *Mb1*$^{Cre/WT}$*Hells*$^{F/WT}$). Unpaired two-tailed *t*-test were performed; ns: non-significant. Bar charts and error bars represent the mean ±SD.

Source data are provided in Source Data File.

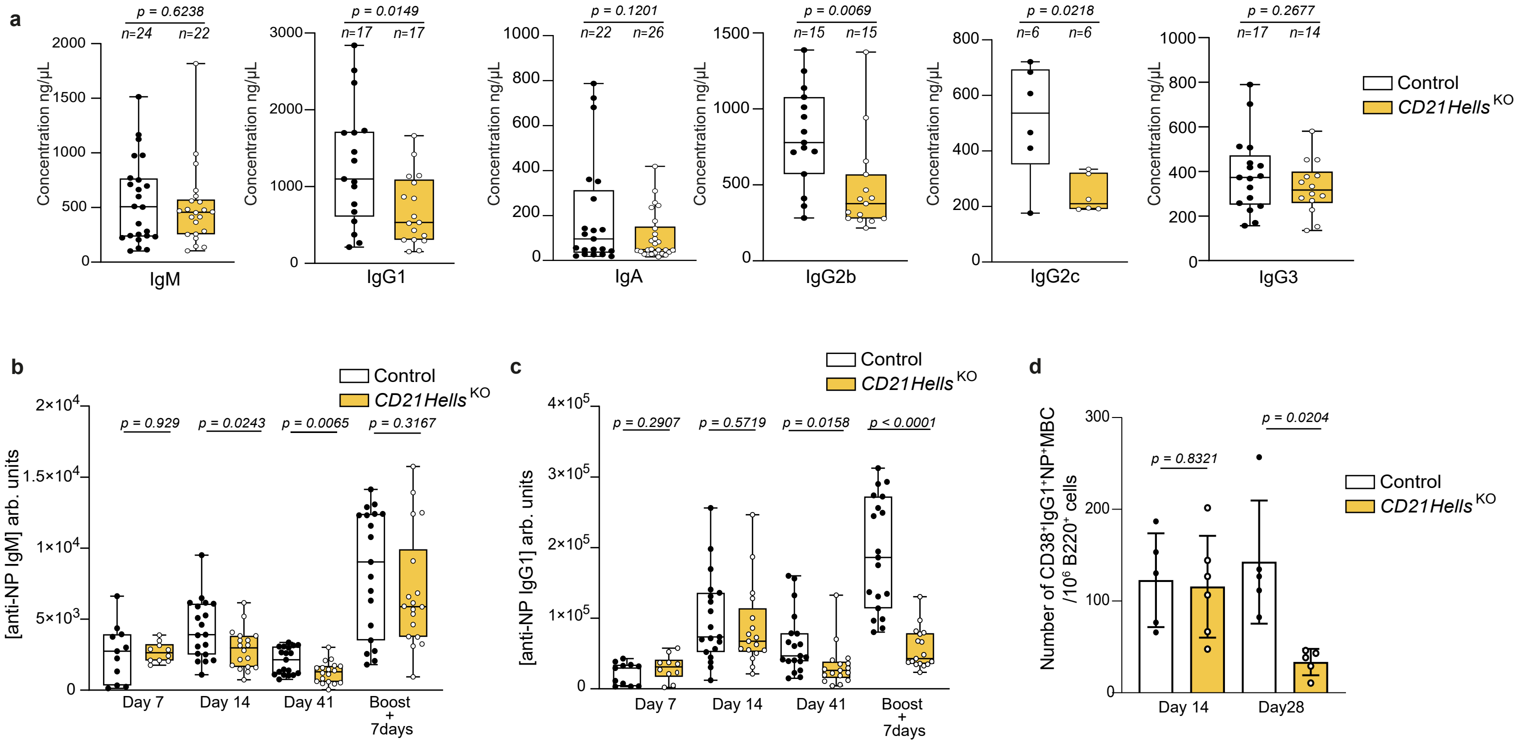

Supplementary Figure 3 – Basal IgG titers and TD humoral response are also affected in *CD21Hells*^{KO} mouse model.

(a) Quantification by ELISA of immunoglobulin titers of non-immunized mice. Box and whisker plots are computed for animals of each genotype for all isotypes. Data were pooled from two independent experiments. *n* for numbers of mice used is indicated in the figure.

(b) and **(c)** Quantification of antigen-specific IgM and IgG1 titers against a TD antigen. *CD21Hells*^{KO} and controls were immunized i.p. with NP-CGG adsorbed on alum and boosted 6 weeks later. Serum was collected at day 7, 14 and 41 after the primary immunization (D7, D14 and D41), and 7 days after the boost. NP-specific IgM and IgG1 titers were quantified by ELISA from *CD21Hells*^{KO} (*n*=17) and control animals (*n*=19), pooled from two independent experiments, except for D7 (*n*=8 and 7 for controls and mutants, respectively).

(c) Quantification of splenic anti-NP-specific MBC (B220^{+}IgM^{neg}IgD^{neg}IgG1^{+}CD38^{+}Gl7^{neg}) in *CD21Hells*^{KO} (*n*=5) and controls (*n*=5) 28 days after immunization with NP-CGG.

Bar charts and error bars represent the mean ±SD. In each boxplot, the bold line in the center of the box indicates the median; and the lower and upper hinges represent, respectively, the first and third quartiles. The whiskers are the minimum and maximum values. Unpaired two-tailed Welch's *t*-test was performed for **(a)**, **(b)**, **(c)** and **(d).** arb.units= arbitrary units. Source data are provided in Source Data File.

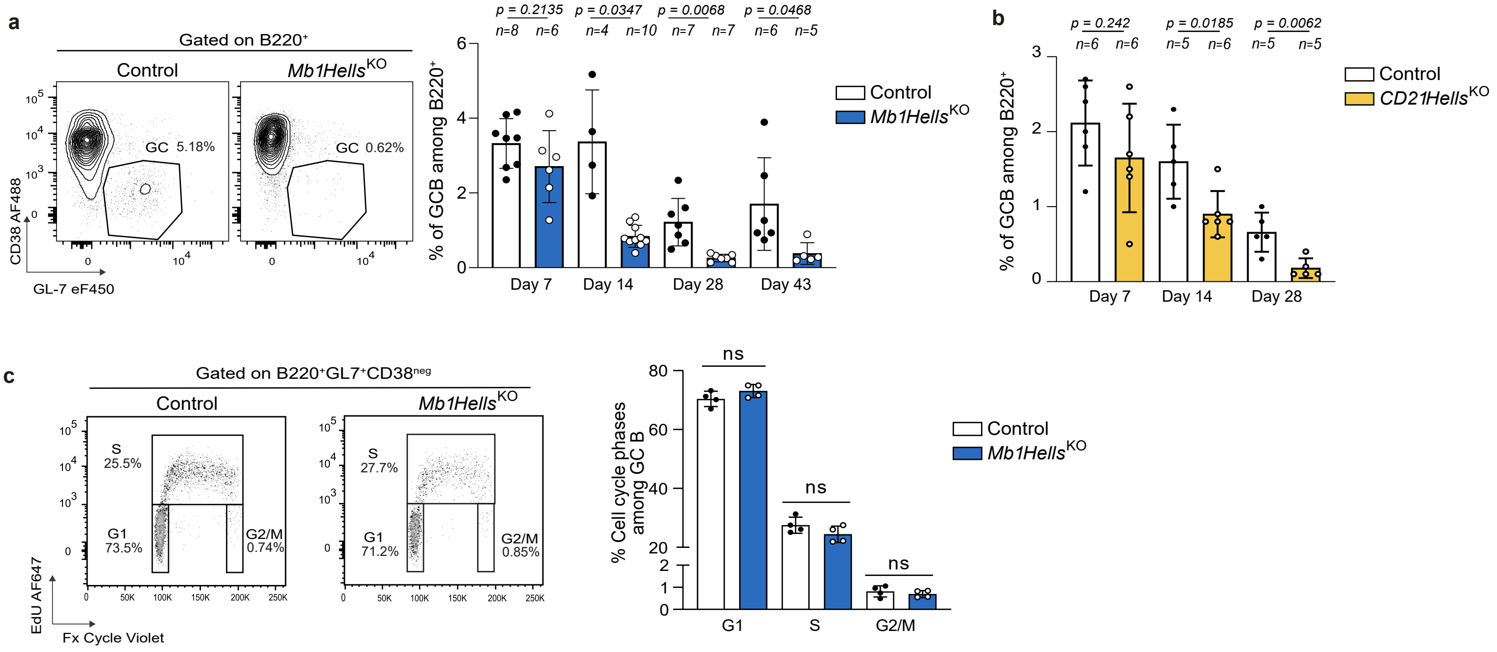

Supplementary Figure 4 – Germinal center B cells devoid of *Hells* form and proliferate normally but collapse prematurely.

(a) Representative Flow Cytometry (FC) plots and quantification of GC B cells ($B220^{+}CD38^{neg}GL7^{hi}$) in *Mb1Hells*^{KO} and controls at different time points after immunization with NP-CGG. *n* for numbers of mice used is indicated in the figure.

(b) Quantification of splenic GC B cell population ($B220^{+}CD38^{lo/neg}GL7^{hi}$) in *CD21Hells*^{KO} and controls, 7, 14 and 28 days after immunization with NP-CGG.

(c) 2D-cell cycle analysis of day 10 GC B cells after *in vivo* EdU labeling. Representative FC plots (left) and quantification of cell cycle phases. Data shown for *n*=4 animals of each genotype and confirmed by independent experiments.

Experiments were performed twice, and one representative experiment is shown. Bar charts and error bars represent the mean ±SD. Unpaired two-tailed *t*-test were performed; ns: non-significant. Source data are provided in Source Data File.

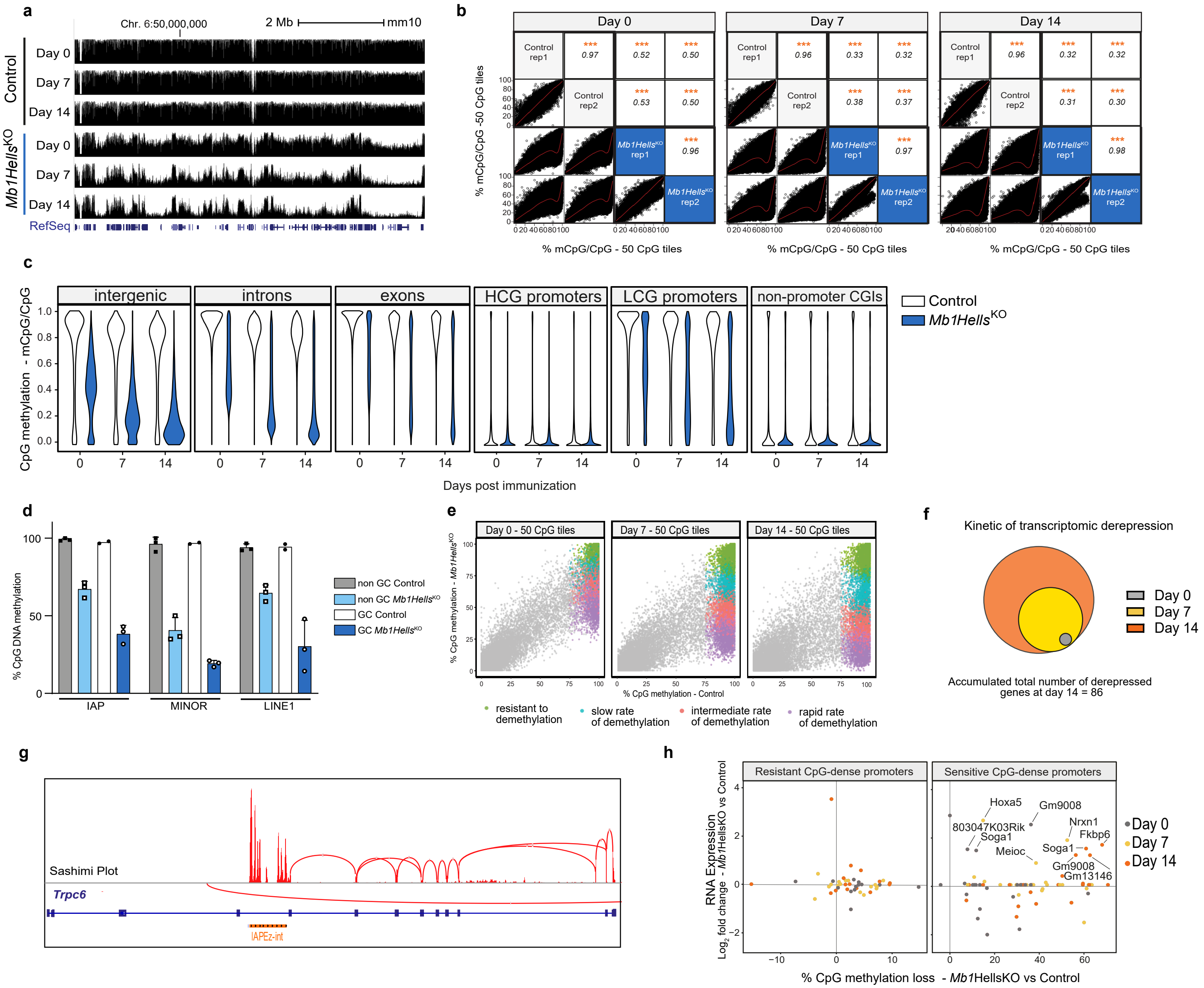

Supplementary Figure 5 – Hells^{KO} GC B cells undergo deep hypomethylation and derepression of repeated non-coding sequences and of a few single-copy genes.

(a) Genome browser view example of global demethylation at a randomly selected genomic region on chromosome 6. Percentage of CpG methylation for 50 CpG genomic tiles is shown for one biological replicate at day 0, day 7, day 14 for control and *Mb1Hells*^{KO} mice.

(b) Scatterplots and Pearson correlation values from comparing percentage of CpG methylation values between each pair of biological replicates for the three time points.

(c) Violin plots displaying methylation levels at single copy sequences. The distribution of mCpG/CpG ratio is shown for 50 CpG genomic tiles, annotated according to Gencode vM25 using preference promoter>exon>intron>intergenic (first three panels); High-CpG density (HCG) promoters, Low-CpG density (LCG) promoters and CpG Islands (CGIs) not overlapping with promoters (panels 4-6).

(d) Percentage of average CpG methylation, inferred from bisulfite sequencing of 9 to 10 representative sequences for IAPEz endogenous retroviruses and LINE-1 retrotransposons amplified from sorted non-GC and GC B cells 10 days after immunization. Bar charts and error bars represent the mean ±SD of 3 independent samples for each genotype.

(e) Kinetics of demethylation at 50 CpG genomic tiles with high CpG density (% CpG > $(0.25)^2$*100). Tiles with WT methylation >75% at all time points were clustered based on demethylation rate in *Mb1Hells*^{KO} using *k*-means clustering with 4 centers.

(f) Circle plot representing the kinetics of derepression of the 86 genes derepressed in day 14 Mb1Hells^{KO} GC B cells (adjusted *p*val <0.05, by Benjamini Hocheberg test).

(g) Sashimi plot for *Trpc6* gene showing internal initiation of transcription from an intronic IAPEz-int copy in one representative day 14 *Mb1Hells*^{KO} GC B cell RNAseq sample.

(h) Relationship between loss of CpG methylation at CpG-dense promoters (% CpG > $(0.25)^2$*100) and log2 expression fold change in *Mb1Hells*^{KO} samples compared to controls at day 0, 7 and 14. Only genes with expression log2 fold change > 0.5 are labeled.

Source data are provided in Source Data File.

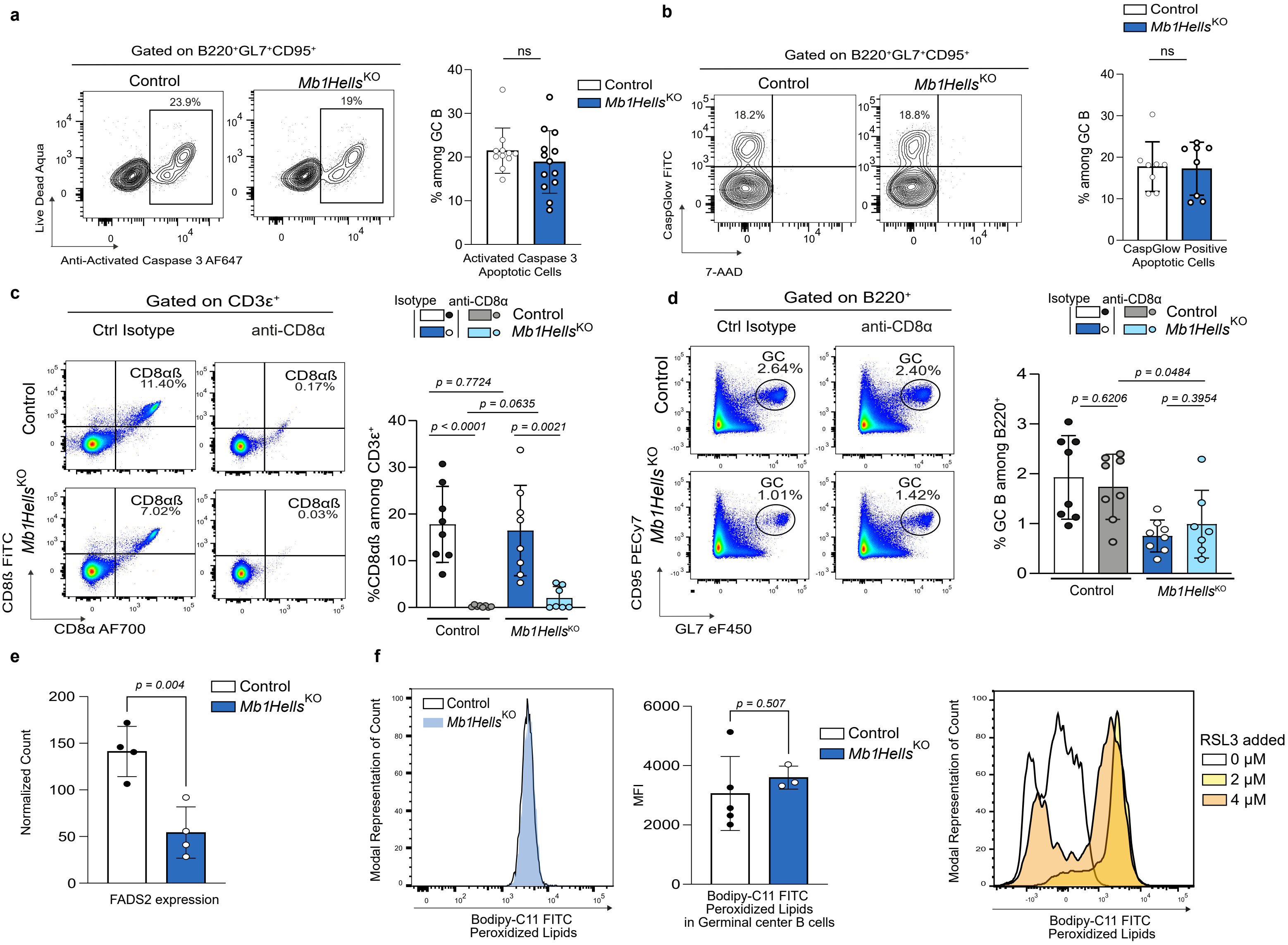

Supplementary Figure 6 – Hells deficient GC B cells do not show increased cell death.

(a) Quantification of apoptosis in day 14 GC B cells by FC, through antibody-detection of active caspase 3.

(b) Quantification of apoptosis in day 10 GC B cells by FC, through detection of active caspases with a pan-caspase FITC-Z-VAD-FMK irreversible inhibitor.

(c) Representative flow cytometry plots and quantification of CD8 T cells ($CD3\varepsilon^{+}B220^{neg}CD8\alpha^{+}CD8\beta^{+}$) in *Mb1Hells*^{KO} and controls 7 days after treatment with depleting anti-CD8α antibody or isotype control (c). Bar charts and error bars represent the mean ±SD of 8 samples for each condition.

(d) GC B cell percentage in immunized mice after depletion of $CD8^{+}$ T cells. Mice immunized with NP-CGG received 7 days later an i.p. injection of 200 µg of depleting anti-CD8α antibody or of an isotype control. Spleens were collected 14 days post-immunization and GC B cells were quantified (*n*= 8 mice for each group). Among the 8 controls treated with anti-CD8α, 2 were *Mb1*$^{Cre/WT}$*Hells*$^{F/WT}$.

(e) Normalized count of FADS2 expression in day 14 GC B cells analyzed by bulk RNAseq (*n*=4 mice for each genotype).

(f) Measurement of ferroptosis by staining peroxidized lipids with Bodipy-C11 in day 10 GC B cells. Quantification of peroxidized lipids was done using flow cytometry, and signal was collected in the FITC and PE channel but quantified in the FITC. (n=3 Mb1HellsKO mice, n=5 controls of which 2 were *Mb1*$^{Cre/WT}$*Hells*$^{F/WT}$). MFI= Mean Fluorescent Intensity. Right panel shows a positive control of lipid peroxidation detection by Bodipy-C11 FITC using 20 hour treatment with RSL3 (0, 2 and 4 µM) on total splenocytes.

Experiments **(a)**, **(b)**, **(c)**, **(d)** were done twice and data were pooled. Experiment **(f)** was done twice, and one representative experiment is shown. Bar charts and error bars represent the mean ± SD. Unpaired two-tailed *t*-test were performed; ns: non-significant. Source data are provided in Source Data File.

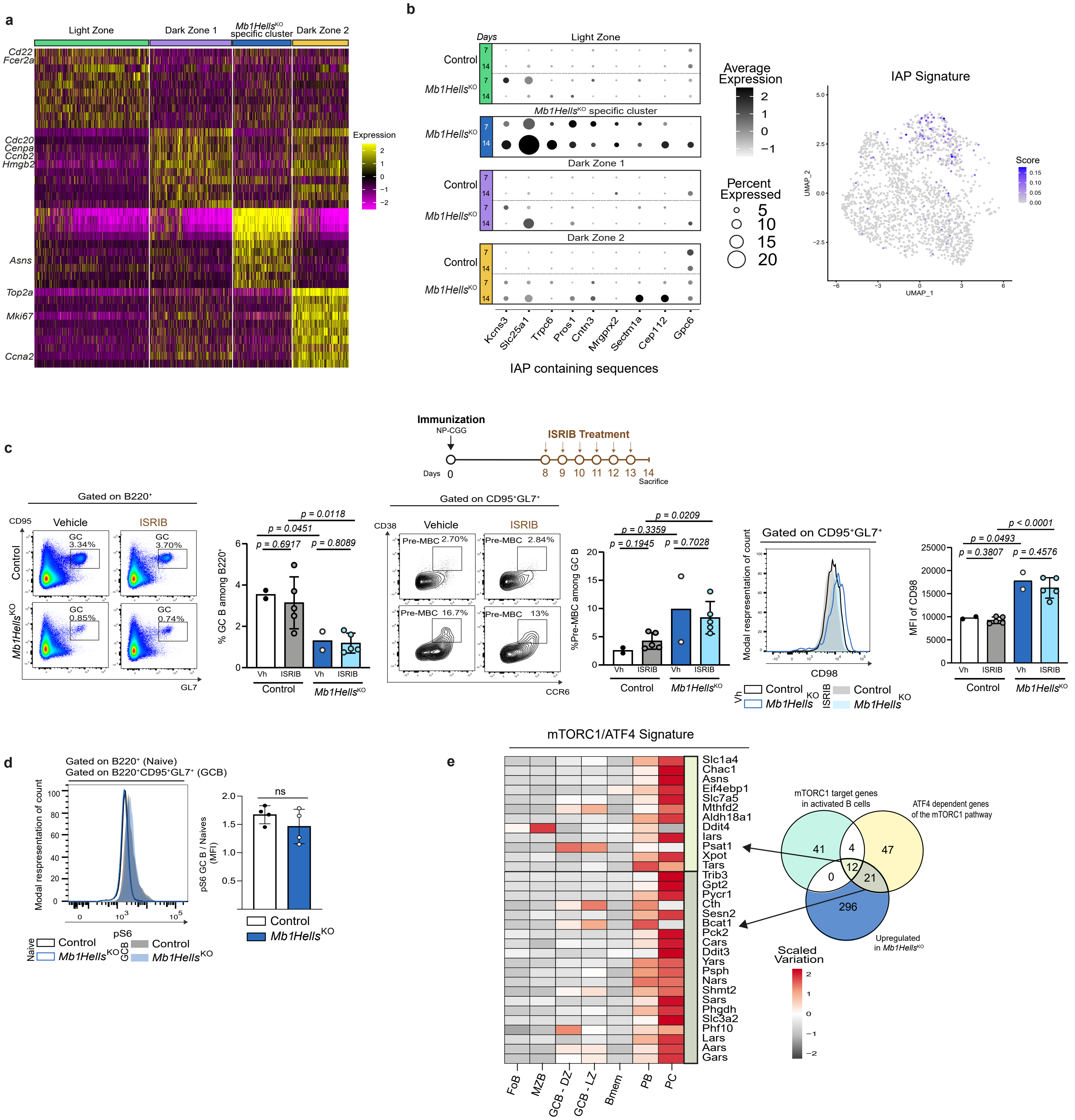

Supplementary Figure 7 – *Hells*^{KO} GC B cells overexpress an mTORC1/ATF4-dependent metabolic pathway upregulated during ASC differentiation, independently from ISR.

(a) Heatmap of each cell's (column) expression of the top ten DEGs per cluster (rows). Log-normalized expression was scaled for each gene. Cluster names are displayed above.

(b) Dotplot and UMAP expression of sequences containing IAP retroviruses by ScRNAseq in D7 and D14 GC B cells from control and *Mb1Hells*^{KO} animals.

(c) Effect of ISR repression through ISRIB treatment on *Mb1Hells*^{KO} and control GC B cells. Experimental setup for ISRIB treatment *in vivo* is shown on the top panel. Representative FC plots and quantification of GC B cells (left) and pre-MBC percentages (middle) after treatment with vehicle or ISRIB, 14 days post immunization. On the right panel, representative CD98 staining of GC B cells and quantification of CD98 geometric MFI (Mean Fluorescent Intensity). Vehicle: *n*=2 mice for each genotype; ISRIB: *n*=5 mice for each genotype.

(d) Representative pS6 staining of naïve and day 14 GC B cells. Bar plots show the ratio of pS6 geometric MFI in GC B and naïve B cells (*n*=4 mice for each genotype). MFI = Mean Fluorescent Intensity.

(e) Heatmap of gene expression within B cell subsets from Immgen database (GSE109125) of the mTORC1/ATF4 signature, which corresponds to genes commonly upregulated in day 14 *Mb1Hells*^{KO} GC B cells, and mTORC1 target genes upregulated in activated B cells (GSE141423) and/or ATF4-dependent targets of mTORC1 (GSE158605).

Experiment **(d)** was performed twice. Bar charts and error bars represent the mean ±SD. Unpaired two-tailed *t*-test were performed for **(c)** and **(d)**; ns: non-significant. Source data are provided in Source Data File.

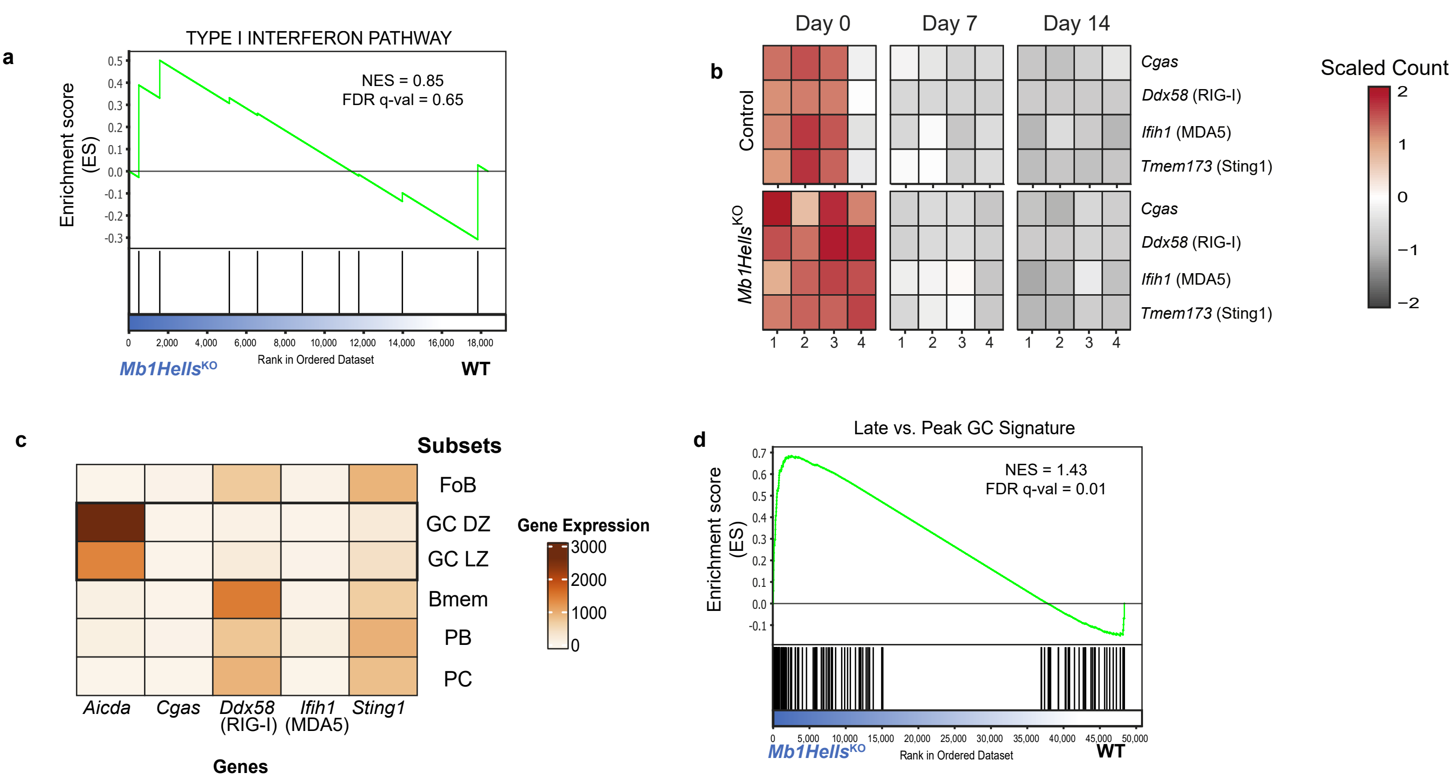

Supplementary Figure 8 – Impact of *Hells* knockout on the expression of type-I interferon and aging-GC signatures.

(a) GSEA showing the absence of enrichment of a type-I IFN signature in day 14 *Mb1Hells*^{KO} GC B cells relative to control day 14 GC B cells. NES= Normalized Enrichment Score, FDR= False Discovery Rate.

(b) Heatmap of the expression of genes involved in cytosolic nucleic acid sensing throughout the GC reaction from bulk-RNAseq data of *n*=4 control and *Mb1Hells*^{KO} mice.

(c) Heatmap of the expression of genes involved in cytosolic nucleic acid sensing throughout late B-cell differentiation. RNAseq data were collected from Immgen database GSE109125.

(d) GSEA showing the enrichment of an aging GC B cell signature in day 14 *Mb1Hells*^{KO} GC B cells vs. day 14 control GC B cells. The aging GC B cell signature is composed of the top-200 genes upregulated in WT day 21 GC B cells relative to WT day 14 GC B cells (GSE 128710). NES= Normalized Enrichment Score, FDR= False Discovery Rate.

Source data are provided in Source Data File.

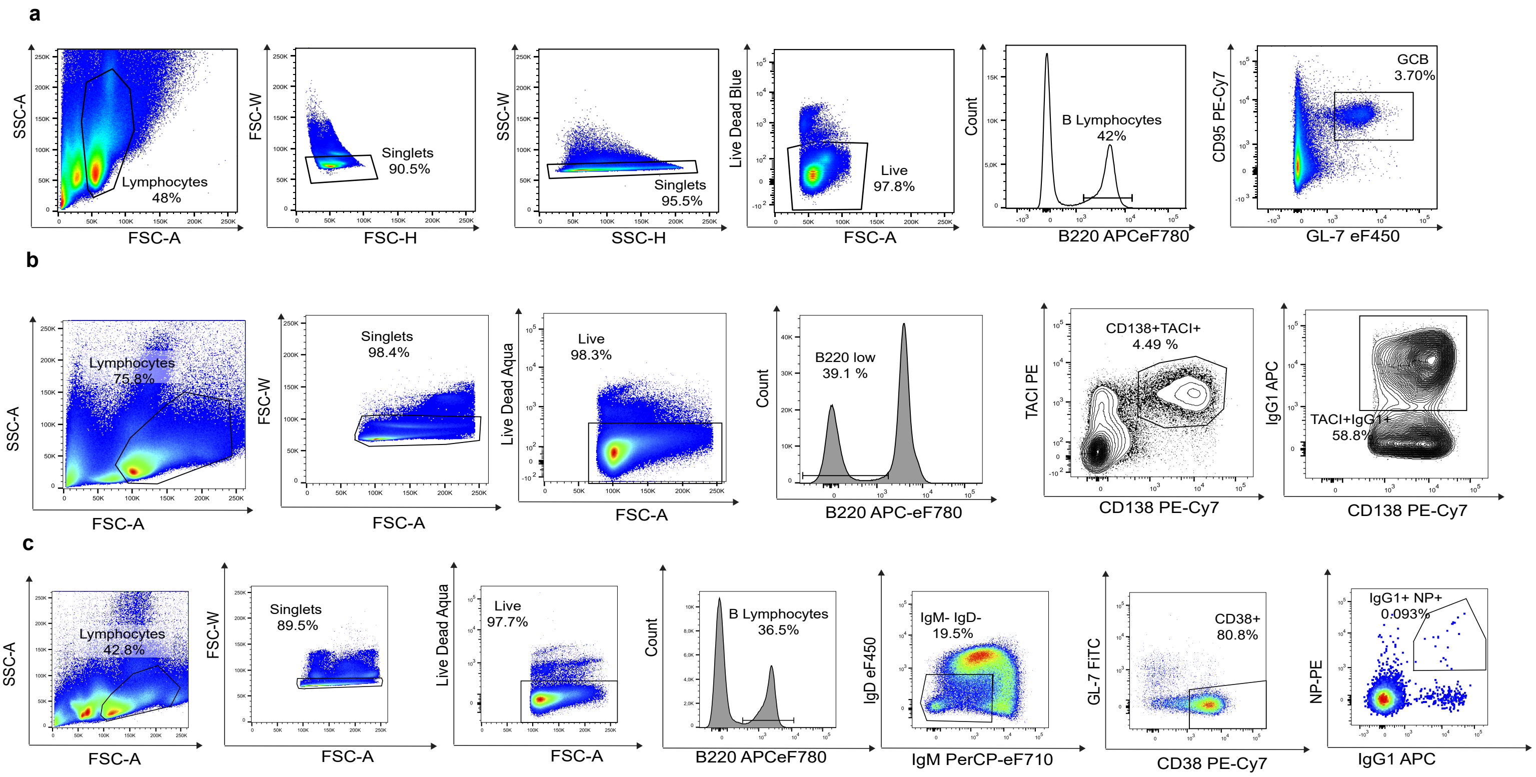

Supplementary Figure 9 - Gating Strategies.
(a) Gating Strategy for Germinal center B cells
(b) Gating Strategy for IgG1+ CD138+ TACI+ Plasma Cells
(c) Gating Strategy for NP+ IgG1+ Memory B cells

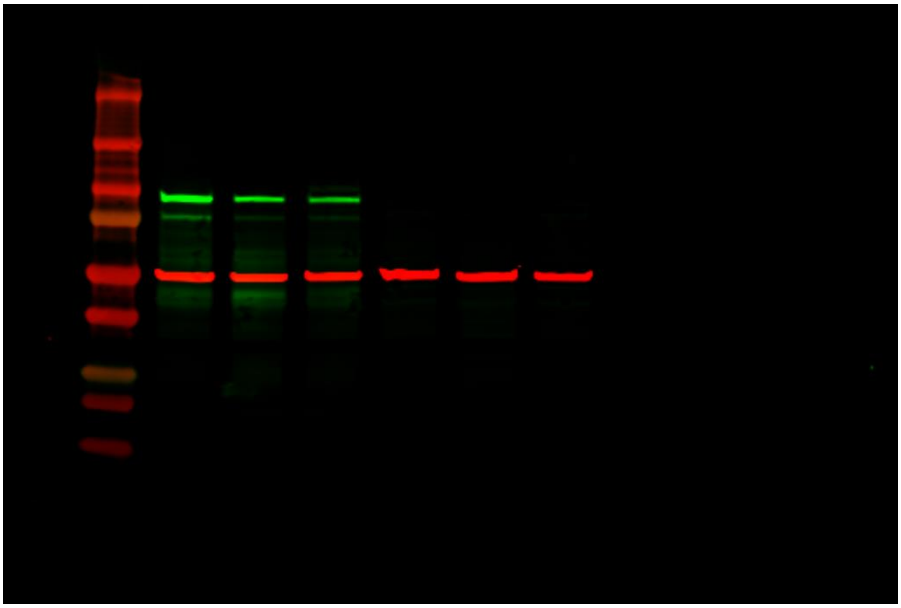

Supplementary Figure 10 – Uncropped western blot shown in Supplementary Figure 2f.

Supplementary Table.1. Table of oligonucleotides.

Genotyping	Mb1Cre	For	CCCTGTGGATGCCACCTC
	Mb1Cre	Rev	GTCCTGGCATCTGTCAGAG
	CD21Cre	For	GGACATGTTCAGGGATCGCCAGGCG
	CD21Cre	Rev	GCATAACCAGTGAAACAGCATTGCTG
	Hells_Flox	For	GTGCATTGGATCCTTTGTGGC
	Hells_Flox	Rev	CATTTCCACTTTAAAACACTCAG
	Hells_Int10	Rev	GGCACATTCAGGACAGTCAG
Hells genomic deletion	HellsEx9	For	GCAGATGAAATGGGTTTGGGA
	HellsEx10	Rev	GGGTTCCATGATACAGCAGAG
	Rev3	For	AGGAAACATGCAATACCTGCGGA
	Rev3	Rev	AGACACACTCTGGTGGTTCGGTG
RT-PCR	mHells-Ex3s	For	CCACTGAAATTCGGTACCGC
	mHells-Ex13AS	Rev	GCAAAGTGGTGCATAAACAACT
	mATF4-BglII	For	GCTCAGATCTACTAGTAACGGCCGCCAG
	mATF4-XhoI	Rev	GCTTCTCGAGCCTATTACGGAACTCTCTTC
RT-qPCR	Hells	For	GCAGATGAAATGGGTTTGGGA
	Hells	Rev	TCATTCGAGATCGCTATGCG
	Gapdh	For	TCACCACCATGGAGAAGGC
	Gapdh	Rev	GCTAAGCAGTTGGTGGTGCA
	Asns	For	GCAGTGTCTGAGTGCGATGAA
	Asns	Rev	TCTTATCGGCTGCATTCCAAAC
	Chac1	For	AGTGTGGAAGCCGGACTTTG
	Chac1	Rev	CACTCGGCCAGGCATCTTGT
	Ubc	For	AGGTCAAACAGGAAGACAGACGTA
	Ubc	Rev	TCACACCCAAGAACAAGCACA
Bisulfite sequencing	MinSat	For	GAGAAATATGGAAAATGATAAAAA
	MinSat	Rev	CATTAATATACACTATTCTACAAATCC
	IAP	For	TTGTGTTTTAAGTGGTAAATAAATAATTTG
	IAP	Rev	CAAAAAAAACACACAAACCAAAAT
	LINE-1	For	TAGGAAATTAGTTTGAATAGGTGAGAGG
	LINE-1	Rev	TCAAACACTATATTACTTTAACAATTCCCA
Somatic Hypermutation	Sc_Vh186.2	For	TTCTTGGCAGCAACAGCTACA
	Sc_Cg1Ext	Rev	GGATCCAGAGTTCCAGGTCACT
	Sc_Cg1Int	Rev	GGAGTTAGTTTGGGCAGCAG
	bulk1_VH186.2	For	CATGGGATGGAGCTGTATCATGC
	bulk1_VH186.2	Rev	CTCACAAGAGTCCGATAGACCCTG
	bulk2_VH186.2	For	GGTGACAATGACATCCACTTTGC
	bulk2_VH186.2	Rev	GACTGTGAGAGTGGTGCCTTG

Supplementary Table.2. Table of antibodies and staining reagents.

Antibodies for flow cytometry					
**Antibody**	**Clone**	**Dilution**	**Source**	**Référence**	**RRID**
anti-human/mouse B220 APC-eF780	RA36B2	1-200	eBioscience	47-0452-82	AB_1518810
anti-mouse B220 AF647	RA3-6B2	1-100	BD Pharmingen	557683	AB_396793
anti-mouse IgG1 APC	X56	1-100	BD Pharmingen	550874	AB_398470
anti-mouse IgM PerCP-eF710	II/41	1-50	eBioscience	46-5790-82	AB_1834435
anti-mouse IgM FITC	Goat anti-mouse	1-100	Southern Biotech	1021-02	AB_2794237
anti-mouse IgD eF450	11.26c	1-100	eBioscience	48-5993-82	AB_1272202
anti-mouse and human Gl7 eF450	GL-7	1-150	eBioscience	48-5902-82	AB_10870775
anti-mouse and human Gl7 FITC	GL-7	1-50	BD Pharmingen	562080	AB_10894953
anti-mouse CD95 PE	Jo2	1-100	BD Pharmingen	561985	AB_10895586
anti-mouse CD95 PE-Cy7	Jo2	1-200	BD Pharmingen	557653	AB_396768
anti-mouse CD38 biotin	90	1-800	BD Pharmingen	553762	AB_395033
anti-mouse CD38 AF488	90	1-200	BioLegend	102714	AB_528796
anti-mouse CD21 PE	7G6	1-100	BD Pharmingen	552957	AB_394532
anti-mouse CD23 PECy7	B3B4	1-100	Sony Biotechnology	1108065	N/A
anti-mouse CD93 APC	AA4.1	1-50	eBioscience	17-5892-82	AB_469466
anti-mouse CD43 BV510	S7	1-100	BD Pharmingen	563206	AB_2738069
anti-mouse CD25 PE	PC61	1-50	BD Pharmingen	561065	AB_10563211
anti-mouse CXCR4 PE/Dazzle 594	L276F12	1-100	BioLegend	146514	AB_2563683
anti-mouse CD86 PE-Cy5	GL1	1-50	eBioscience	15-0862-82	AB_468778
anti-mouse TACI PE	eBio8F10-3	1-100	eBioscience	12-5942-81	AB_837121
anti-mouse CD138 PE-Cy7	281-2	1-50	BioLegend	142514	AB_2562198
anti-mouse CD19 PerCP-Cy5-5	1D3	1-100	BD Pharmingen	551001	AB_394004
anti-human CD3 APC-H7	SK7	1-100	BD Pharmingen	560176	AB_1645475
anti-human CD14 APC-H7	MφP9	1-100	BD Pharmingen	560180	AB_1645464
anti-human CD16 APC-H7	3G8	1-100	BD Pharmingen	560715	AB_1727432
anti-human CD19 V500	HIB19	1-100	BD Pharmingen	561121	AB_10562391
anti-human CD38 PerCPCy5-5	HIT2	1-100	Sony Biotechnology	2117610	N/A
anti-human CD24 PE	ML5	1-100	BioLegend	311106	AB_314855
anti-human CD27 APC	M-T271	1-100	BD Pharmingen	561400	AB_10645790
anti-human IgD PE-CF594	IA6-2	1-100	BD Pharmingen	562540	AB_11153129
anti-human IgM BV605	MHM-88	1-100	Sony Biotechnology	2172615	N/A
Anti-mouse CD8a AF700	53-6.7	1-150	eBioscience	56-0081-82	AB_494005
Anti-mouse CD8b.2 FITC	53-5.8	1-200	BioLegend	140404	AB_10643587
Anti-mouse CD3e PE	145-2C11	1-100	BD Pharmingen	553063	AB_394596
PNA Biotin	B-1075	1-10000	Vector Laboratories	B-1075-5	N/A
Anti-mouse CCR6 BV785	29-2L17	1-200	BioLegend	129823	AB_2715923
Anti-mouse CD98 PE	RL388	1-200	BioLegend	128207	AB_1186107
Anti-mouse pS6 AF488	D57.2.2E	1-100	Cell Signaling Technology	#4803	AB_916158
Rabbit IgG isotype control AF488	DA1E	1-100	Cell Signaling Technology	2975	AB_10699151
Anti-activated Caspase 3 AF647	C92-605	1-20	BD Pharmingen	560626	AB_1727414
Rabbit anti-mouse ATF4	D4B8	1-100	Cell Signaling Technology	11815	AB_2616025
Anti-rabbit IgG (H+L), F(ab')2 AF647	Goat, polyclonal	1-500	Cell Signaling Technology	4414	AB_10693544
Streptavidin PE-Cy7		1-700	Sony Biotechnology	2626030	N/A
**Staining reagents for flow cytometry**					
7-AAD		1-100	BioLegend	420403	N/A
Sytox Blue		1µL.mL^{-1}	Invitrogen	S34857	N/A
Live Dead Aqua		1µL.mL^{-1}	Invitrogen	L34965	N/A
Live Dead Blue		1µL.mL^{-1}	Invitrogen	L23105	N/A
CaspGLOW			Invitrogen	88-7003-42	N/A

<table>
<tr><td>BODIPY-C11</td><td></td><td>2 µM</td><td>Invitrogen</td><td>D3861</td><td>N/A</td></tr>
<tr><td colspan="6">Antibodies for in vivo depletion</td></tr>
<tr><td>Depleting Anti-mouse CD8a</td><td>2.43</td><td>200 µg</td><td>BioXCell</td><td>BE0061</td><td>N/A</td></tr>
<tr><td>Depleting Rat IgG2b control Isotype</td><td>LTF-2</td><td>200 µg</td><td>BioXCell</td><td>BE0090</td><td>N/A</td></tr>
<tr><td colspan="6">Antibodies for western-blot</td></tr>
<tr><td>anti-HELLS</td><td>Rabbit, polyclonal</td><td>1-2000</td><td>ProteinTech</td><td>11955-1-AP</td><td>AB_2117529</td></tr>
<tr><td>Mouse monoclonal anti-α Tubulin</td><td>DM1A</td><td>1-5000</td><td>Sigma</td><td>T6199</td><td>AB_2617116</td></tr>
<tr><td>IRDye® 680RD Goat anti-Mouse IgG</td><td>Goat, polyclonal</td><td>1-15000</td><td>LI-COR Biosciences</td><td>926-68070</td><td>AB_10956588</td></tr>
<tr><td>IRDye® 800CW Goat anti-Rabbit</td><td>Goat, polyclonal</td><td>1-15000</td><td>LI-COR Biosciences</td><td>926-32211</td><td>AB_621843</td></tr>
<tr><td colspan="6">Antibodies for ELISA and ELISPOT</td></tr>
<tr><td>Goat anti-mouse Ig</td><td>Goat, polyclonal</td><td>10 µg.mL^{-1}</td><td>SouthernBiotech</td><td>1010-01</td><td>AB_2794121</td></tr>
<tr><td>HRP-Goat anti-mouse IgM</td><td rowspan="6">Goat, polyclonal</td><td rowspan="6">1-2000</td><td rowspan="6">SouthernBiotech</td><td rowspan="6">5300-05B</td><td rowspan="6">AB_2794201</td></tr>
<tr><td>HRP-Goat anti-mouse IgG1</td></tr>
<tr><td>HRP-Goat anti-mouse IgG2b</td></tr>
<tr><td>HRP-Goat anti-mouse IgG2c</td></tr>
<tr><td>HRP-Goat anti-mouse IgG3</td></tr>
<tr><td>HRP-Goat anti-mouse IgA</td></tr>
<tr><td>Purified mouse IgM</td><td rowspan="6"></td><td rowspan="6"></td><td rowspan="6">SouthernBiotech</td><td rowspan="6">5300-01B</td><td rowspan="6">AB_2796077</td></tr>
<tr><td>Purified mouse IgG1</td></tr>
<tr><td>Purified mouse IgG2b</td></tr>
<tr><td>Purified mouse IgG2c</td></tr>
<tr><td>Purified mouse IgG3</td></tr>
<tr><td>Purified mouse IgA</td></tr>
</table>
