# Supplementary material for: Germinal center output is sustained by HELLS-dependent DNA-methylation-maintenance in B cells
Source: Nat Commun. 2023 Sep 14;14:5695. doi: 10.1038/s41467-023-41317-3 (PMC10502085; doi:10.1038/s41467-023-41317-3)
Supplement: Supplementary file 3 — Reporting Summary [file 41467_2023_41317_MOESM3_ESM.pdf]

nature portfolio

Corresponding author(s): Sébastien Storck

Last updated by author(s): Aug 15, 2023

Reporting Summary

Nature Portfolio wishes to improve the reproducibility of the work that we publish. This form provides structure for consistency and transparency in reporting. For further information on Nature Portfolio policies, see our Editorial Policies and the Editorial Policy Checklist.

Statistics

For all statistical analyses, confirm that the following items are present in the figure legend, table legend, main text, or Methods section.

n/a	Confirmed	
☐	☒	The exact sample size (*n*) for each experimental group/condition, given as a discrete number and unit of measurement
☐	☒	A statement on whether measurements were taken from distinct samples or whether the same sample was measured repeatedly
☐	☒	The statistical test(s) used AND whether they are one- or two-sided<br>*Only common tests should be described solely by name; describe more complex techniques in the Methods section.*
☒	☐	A description of all covariates tested
☐	☒	A description of any assumptions or corrections, such as tests of normality and adjustment for multiple comparisons
☐	☒	A full description of the statistical parameters including central tendency (e.g. means) or other basic estimates (e.g. regression coefficient) AND variation (e.g. standard deviation) or associated estimates of uncertainty (e.g. confidence intervals)
☐	☒	For null hypothesis testing, the test statistic (e.g. *F*, *t*, *r*) with confidence intervals, effect sizes, degrees of freedom and *P* value noted<br>*Give P values as exact values whenever suitable.*
☒	☐	For Bayesian analysis, information on the choice of priors and Markov chain Monte Carlo settings
☒	☐	For hierarchical and complex designs, identification of the appropriate level for tests and full reporting of outcomes
☐	☒	Estimates of effect sizes (e.g. Cohen's *d*, Pearson's *r*), indicating how they were calculated

Our web collection on statistics for biologists contains articles on many of the points above.

Software and code

Policy information about availability of computer code

Data collection	Flow Data were collected using BD Diva Software version (X) on BD FACS Canto II or LSRFortessa. Sorting flow cytometry was performed using BD FACS Aria III and BD FACS Aria II SORP<br>ELISA data were collected on SkanIt Software 5.0 for Microplate Readers RE<br>ELISPOT data were collected using AID software (version 3.5: Autoimmun Diagnostika)<br>Western blot was acquired with an Odyssey CLx Infrared Imaging device<br>Real-time PCR data were acquired with a CFX96 Touch Real-Time PCR Dectection System (Bio-Rad)<br>RNA integrity was monitored with Fragment Analyzer (Agilent Technologies)
Data analysis	Flow cytometry data analysis was performed using FlowJo (Treestar) software version 10.8.1<br>Statistical Analysis was performed using Graphpad Prism version 9.4.1<br>Sequencing data were Analyzed on CodonCode (v10.0.02) (CodonCode Corporation)<br>Bulk RNAseq FASTQ files were mapped to the ENSEMBL Mouse GRCm38/mm10 reference using HISTA2 (v2.1.0; https://daehwankimlab.github.io/hisat2/); read count normalization and groups comparisons were performed by DESeq2 (v1.24.0), on R version 3.6.2<br>Heatmaps were generated with pheatmap Package on R version 3.6.2<br>Data Set Enrichment was performed using GSEA v4.2.3 (Broad Institute)<br>Single cell RNAseq data were performed on R version 4.1.2 using Seurat Package v4.1.1<br>EM-seq data paired-end analysis was performed using FastQC v0.11.8, raw reads were trimmed to remove both poor-quality calls and adapters using Trim Galore (v0.6.4,), trimmed reads were aligned to GRCm38 mouse genome assembly with Bismark v0.22.3. Methylation quantitation over tiles was performed in a custom R script using function ‘regionCounts’ from bioconductor package methylKit v1.10.0 : https://zenodo.org/record/8208644, https://github.com/boulardlab/BS_EMSeq. CustViolin plots were generated with bioconductor package genomation v1.16.0

For RNA-seq analysis of Transposable Elements (TE), FASTQ files were analyzed using FastQC (v0.11.8), raw data were trimmed using Trim Galore (v0.6.4), and trimmed reads were aligned to GRCm38 mouse genome with STAR v2.7.5c. Reads from pairs where both mates were completely included into a repetetive element were selected with a custom code : https://zenodo.org/record/8208681, https://github.com/boulardlab/BS_RNASeq. Differential expression of TE was analyzed using bioconductor package DESeq2 v1.24.0
Visualization of IAP-ez was done with Integrative Genomics Viewer software from Broad Institute (IGV, version 2.8.2)
All original code has been deposited at GitHub and is publicly available under the DOIs: https://zenodo.org/record/8208644 and https://zenodo.org/record/8208681 and the following links: https://github.com/boulardlab/BS_RNASeq and https://github.com/boulardlab/BS_EMSeq.

For manuscripts utilizing custom algorithms or software that are central to the research but not yet described in published literature, software must be made available to editors and reviewers. We strongly encourage code deposition in a community repository (e.g. GitHub). See the Nature Portfolio guidelines for submitting code & software for further information.

Data

Policy information about availability of data

All manuscripts must include a data availability statement. This statement should provide the following information, where applicable:

- Accession codes, unique identifiers, or web links for publicly available datasets
- A description of any restrictions on data availability
- For clinical datasets or third party data, please ensure that the statement adheres to our policy

The bulk RNAseq data generated in this study have been deposited in the Array Express database under accession code E-MTAB-12638 (https://www.ebi.ac.uk/biostudies/arrayexpress/studies/E-MTAB-12638). The single cell RNAseq data generated in this study have been deposited in the Array Express database under accession code E-MTAB-12499 (https://www.ebi.ac.uk/biostudies/arrayexpress/studies/E-MTAB-12499). The EM-seq data generated in this study have been deposited in the Array Express database under accession code E-MTAB-12609 (https://www.ebi.ac.uk/biostudies/arrayexpress/studies/E-MTAB-12609).
Other RNAseq data used in this study are available in the GEO database under accession codes :
• GSE109125 (https://www.ncbi.nlm.nih.gov/geo/query/acc.cgi?acc=GSE109125)
• GSE158605 (https://www.ncbi.nlm.nih.gov/geo/query/acc.cgi?acc=GSE158605)
• GSE141423 (https://www.ncbi.nlm.nih.gov/geo/query/acc.cgi?acc=GSE141423)
• GSE60927 (https://www.ncbi.nlm.nih.gov/geo/query/acc.cgi?acc=GSE60927)
• GSE115656 https://www.ncbi.nlm.nih.gov/geo/query/acc.cgi?acc=GSE115656)
• GSE89897 (https://www.ncbi.nlm.nih.gov/geo/query/acc.cgi?acc=GSE89897)
• GSE110669 (https://www.ncbi.nlm.nih.gov/geo/query/acc.cgi?acc=GSE110669)
• GSE128710 (https://www.ncbi.nlm.nih.gov/geo/query/acc.cgi?acc=GSE128710).
Mus musculus genome assembly mm10 is available through the hyperlink https://www.ncbi.nlm.nih.gov/datasets/genome/GCF_000001635.20/.
Repeat Library 20140131 (mm10, Dec 2011) : http://repeatmasker.org.
All other original data from this study are included in the text. Source data are provided with this paper.

Human research participants

Policy information about studies involving human research participants and Sex and Gender in Research.

Reporting on sex and gender	Spleen samples were obtained from one male and one female pediatric patient.
Population characteristics	Both pediatric patients underwent splenectomy to treat sickle cell disease. Age : 4.5 and 6 years old.
Recruitment	The study on pediatric splenic samples was conducted in compliance with the Declaration of Helsinki. Parents from pediatric patients provided written informed consent before the collection of splenic samples.
Ethics oversight	Spleen samples were conserved and prepared with the authorization of the Comité de Protection des Personnes Ile de France II (DC-2008-448).

Note that full information on the approval of the study protocol must also be provided in the manuscript.

Field-specific reporting

Please select the one below that is the best fit for your research. If you are not sure, read the appropriate sections before making your selection.

☒ Life sciences ☐ Behavioural & social sciences ☐ Ecological, evolutionary & environmental sciences

For a reference copy of the document with all sections, see nature.com/documents/nr-reporting-summary-flat.pdf

Life sciences study design

All studies must disclose on these points even when the disclosure is negative.

Sample size	No statistical methods were used to determine sample size. Sample size was determined to be adequate based on reproducibility between independent experiments, and common practice in the field of B-cell biology, such as those used in Litzler et al., Nat Comm 2019 10(1):22 for

	a similar work. RNA from 4 different mice of each genotype were used for each time point, as recommended (n≥3, Conesa et al, Genome Biol. 2016 17:13)
Data exclusions	No data were excluded from the analysis.
Replication	At least two independent experiments were performed for each figure. The reproductibility was based on the magnitude and consistency of measurable differences between samples.
Randomization	For specific DNMT1 inhibitor, mice were allocated randomly and age-matched and sex-matched animals were distributed equally in groups. For other experiments, no random allocation was performed as mice were genotyped prior to experiments.
Blinding	Experiments were not performed in a blinded fashion as each sample needed to be double checked for genotype.

Reporting for specific materials, systems and methods

We require information from authors about some types of materials, experimental systems and methods used in many studies. Here, indicate whether each material, system or method listed is relevant to your study. If you are not sure if a list item applies to your research, read the appropriate section before selecting a response.

Materials & experimental systems

n/a	Involved in the study
☐	☒ Antibodies
☐	☒ Eukaryotic cell lines
☒	☐ Palaeontology and archaeology
☐	☒ Animals and other organisms
☒	☐ Clinical data
☒	☐ Dual use research of concern

Methods

n/a	Involved in the study
☒	☐ ChIP-seq
☐	☒ Flow cytometry
☒	☐ MRI-based neuroimaging

Antibodies

Antibodies used

Antibody, Clone, Dilution, Source, Reference, RRID
anti-human/mouse B220 APC-eF780, RA36B2, 1-200, eBioscience, 47-0452-82, AB_1518810
anti-mouse B220 AF647, RA3-6B2, 1-100, BD Pharmingen, 557683, AB_396793
anti-mouse IgG1 APC, X56, 1-100, BD Pharmingen, 550874, AB_398470
anti-mouse IgM PerCP-eF710, II/41, 1-50, eBioscience, 46-5790-82, AB_1834435
anti-mouse IgM FITC, Goat anti-mouse, 1-100, Southern Biotech, 1021-02, AB_2794237
anti-mouse IgD eF450, 11.26c, 1-100, eBioscience, 48-5993-82, AB_1272202
anti-mouse and human Gl7 eF450, GL-7, 1-150, eBioscience, 48-5902-82, AB_10870775
anti-mouse and human Gl7 FITC, GL-7, 1-50, BD Pharmingen, 562080, AB_10894953
anti-mouse CD95 PE, Jo2, 1-100, BD Pharmingen, 561985, AB_10895586
anti-mouse CD95 PE-Cy7, Jo2, 1-200, BD Pharmingen, 557653, AB_396768
anti-mouse CD38 biotin, 90, 1-800, BD Pharmingen, 553762, AB_395033
anti-mouse CD38 AF488, 90, 1-200, BioLegend, 102714, AB_528796
anti-mouse CD21 PE, 7G6, 1-100, BD Pharmingen, 552957, AB_394532
anti-mouse CD23 PECy7, B3B4, 1-100, Sony Biotechnology, 1108065, N/A
anti-mouse CD93 APC, AA4.1, 1-50, eBioscience, 17-5892-82, AB_469466
anti-mouse CD43 BV510, S7, 1-100, BD Pharmingen, 563206, AB_2738069
anti-mouse CD25 PE, PC61, 1-50, BD Pharmingen, 561065, AB_10563211
anti-mouse CXCR4 PE/Dazzle 594, L276F12, 1-100, BioLegend, 146514, AB_2563683
anti-mouse CD86 PE-Cy5, GL1, 1-50, eBioscience, 15-0862-82, AB_468778
anti-mouse TACI PE, eBio8F10-3, 1-100, eBioscience, 12-5942-81, AB_837121
anti-mouse CD138 PE-Cy7, 281-2, 1-50, BioLegend, 142514, AB_2562198
anti-mouse CD19 PerCP-Cy5-5, 1D3, 1-100, BD Pharmingen, 551001, AB_394004
anti-human CD3 APC-H7, SK7, 1-100, BD Pharmingen, 560176, AB_1645475
anti-human CD14 APC-H7, MφP9, 1-100, BD Pharmingen, 560180, AB_1645464
anti-human CD16 APC-H7, 3G8, 1-100, BD Pharmingen, 560715, AB_1727432
anti-human CD19 V500, HIB19, 1-100, BD Pharmingen, 561121, AB_10562391
anti-human CD38 PerCPCy5-5, HIT2, 1-100, Sony Biotechnology, 2117610, N/A
anti-human CD24 PE, ML5, 1-100, BioLegend, 311106, AB_314855
anti-human CD27 APC, M-T271, 1-100, BD Pharmingen, 561400, AB_10645790
anti-human IgD PE-CF594, IA6-2, 1-100, BD Pharmingen, 562540, AB_11153129
anti-human IgM BV605, MHM-88, 1-100, Sony Biotechnology, 2172615, N/A
anti-mouse CD8a AF700, 53-6.7, 1-150, eBioscience, 56-0081-82, AB_494005
anti-mouse CD8b.2 FITC, 53-5.8, 1-200, BioLegend, 140404, AB_10643587
anti-mouse CD3e PE, 145-2C11, 1-100, BD Pharmingen, 553063, AB_394596
PNA Biotin, B-1075, 1-10000, Vector Laboratories, B-1075-5, N/A
anti-mouse CCR6 BV785, 29-2L17, 1-200, BioLegend, 129823, AB_2715923
anti-mouse CD98 PE, RL388 , 1-200, BioLegend, 128207, AB_1186107

anti-mouse pS6 AF488, D57.2.2E, 1-100, Cell Signaling Technology, #4803, AB_916158
Rabbit IgG isotype control AF488 , DA1E , 1-100, Cell Signaling Technology, 2975, AB_10699151
anti-activated Caspase 3 AF647, C92-605, 1-20, BD Pharmingen, 560626, AB_1727414
Rabbit anti-mouse ATF4 , D4B8, 1-100, Cell Signaling Technology, 11815, AB_2616025
anti-rabbit IgG (H+L), F(ab')2 AF647, Goat, polyclonal, 1-500, Cell Signaling Technology, 4414, AB_10693544
Streptavidin PE-Cy7, N/A, 1-700, Sony Biotechnology, 2626030, N/A
7-AAD, N/A , 1-100, BioLegend, 420403, N/A
Sytox Blue, N/A, 1-1000, Invitrogen, S34857, N/A
Live Dead Aqua, N/A, 1-1000, Invitrogen, L34965, N/A
Live Dead Blue, N/A, 1-1000, Invitrogen, L23105, N/A
CaspGLOW, N/A, Invitrogen, 88-7003-42, N/A
BODIPY-C11, N/A, 2 µM, Invitrogen, D3861, N/A
Depleting Anti-mouse CD8a, 2.43, 200 µg, BioXCell, BE0061, N/A
Depleting Rat IgG2b control Isotype, LTF-2, 200 µg, BioXCell, BE0090, N/A
anti-HELLS, Rabbit, polyclonal, 1-2000, ProteinTech , 11955-1-AP, AB_2117529
Mouse monoclonal anti-α Tubulin, DM1A, 1-5000, Sigma, T6199, AB_2617116
IRDye® 680RD Goat anti-Mouse IgG, Goat, polyclonal, 1-15000, LI-COR Biosciences, 926-68070, AB_10956588
IRDye® 800CW Goat anti-Rabbit, Goat, polyclonal, 1-15000, LI-COR Biosciences, 926-32211, AB_621843
Goat anti-mouse Ig, Goat, polyclonal, 10 µg.mL-1, SouthernBiotech, 1010-01, AB_2794121
HRP-Goat anti-mouse IgM, N/A, 1-2000, SouthernBiotech, 5300-05B, AB_2794201
HRP-Goat anti-mouse IgG1, N/A, 1-2000, SouthernBiotech, 5300-05B, AB_2794201
HRP-Goat anti-mouse IgG2b, N/A, 1-2000, SouthernBiotech, 5300-05B, AB_2794201
HRP-Goat anti-mouse IgG2c,N/A , 1-2000, SouthernBiotech, 5300-05B, AB_2794201
HRP-Goat anti-mouse IgG3, N/A, 1-2000, SouthernBiotech, , 5300-05B, AB_2794201
HRP-Goat anti-mouse IgA, N/A, 1-2000, SouthernBiotech, 5300-05B, AB_2794201
Purified mouse IgM, N/A, N/A, SouthernBiotech, 5300-01B, AB_2796077
Purified mouse IgG1, N/A, N/A, SouthernBiotech, 5300-01B, AB_2796077
Purified mouse IgG2b, N/A, N/A, SouthernBiotech, 5300-01B, AB_2796077
Purified mouse IgG2c, N/A, N/A, SouthernBiotech, 5300-01B, AB_2796077
Purified mouse IgG3, N/A, N/A, SouthernBiotech, 5300-01B, AB_2796077
Purified mouse IgA, N/A, N/A, SouthernBiotech, 5300-01B, AB_2796077

Validation

All the antibodies used were validated by manufacturers on their official website. All antibodies and clones used are common and described in the literature.
Antibody, Clone, Source, Reference, RRID, validation
• anti-human/mouse B220 APC-eF780, RA36B2, eBioscience, 47-0452-82, AB_1518810, https://www.thermofisher.com/antibody/product/CD45R-B220-Antibody-clone-RA3-6B2-Monoclonal/47-0452-82
• anti-mouse B220 AF647, RA3-6B2, BD Pharmingen, 557683, AB_396793, https://www.bdbiosciences.com/en-fr/products/reagents/flow-cytometry-reagents/research-reagents/single-color-antibodies-ruo/alexa-fluor-647-rat-anti-mouse-cd45r.557683
• anti-mouse IgG1 APC, X56, BD Pharmingen, 550874, AB_398470, https://www.bdbiosciences.com/en-fr/products/reagents/flow-cytometry-reagents/research-reagents/single-color-antibodies-ruo/apc-rat-anti-mouse-igg1.550874
• anti-mouse IgM PerCP-eF710, II/41, eBioscience, 46-5790-82, AB_1834435, https://www.thermofisher.com/antibody/product/IgM-Antibody-clone-II-41-Monoclonal/46-5790-82
• anti-mouse IgM FITC, Goat anti-mouse, Southern Biotech, 1021-02, AB_2794237, https://www.southernbiotech.com/goat-anti-mouse-igm-fitc-1021-02
• anti-mouse IgD eF450, 11.26c, eBioscience, 48-5993-82, AB_1272202, https://www.thermofisher.com/antibody/product/IgD-Antibody-clone-11-26c-11-26-Monoclonal/48-5993-82
• anti-mouse and human Gl7 eF450, GL-7, eBioscience, 48-5902-82, AB_10870775, https://www.thermofisher.com/antibody/product/GL7-Antibody-clone-GL-7-GL7-Monoclonal/48-5902-82
• anti-mouse and human Gl7 FITC, GL-7, BD Pharmingen, 562080, AB_10894953, https://www.bdbiosciences.com/en-fr/products/reagents/flow-cytometry-reagents/research-reagents/single-color-antibodies-ruo/fitc-rat-anti-mouse-t-and-b-cell-activation-antigen.562080
• anti-mouse CD95 PE, Jo2, BD Pharmingen, 561985, AB_10895586, https://www.bdbiosciences.com/en-fr/products/reagents/flow-cytometry-reagents/research-reagents/single-color-antibodies-ruo/pe-hamster-anti-mouse-cd95.561985
• anti-mouse CD95 PE-Cy7, Jo2, BD Pharmingen, 557653, AB_396768, https://www.bdbiosciences.com/en-fr/products/reagents/flow-cytometry-reagents/research-reagents/single-color-antibodies-ruo/pe-cy-7-hamster-anti-mouse-cd95.557653
• anti-mouse CD38 biotin, 90, BD Pharmingen, 553762, AB_395033, https://www.bdbiosciences.com/en-fr/products/reagents/flow-cytometry-reagents/research-reagents/single-color-antibodies-ruo/biotin-rat-anti-mouse-cd38.553762
• anti-mouse CD38 AF488, 90, BioLegend, 102714, AB_528796, https://www.biolegend.com/en-us/products/alexa-fluor-488-anti-mouse-cd38-antibody-3313
• anti-mouse CD21 PE, 7G6, BD Pharmingen, 552957, AB_394532, https://www.bdbiosciences.com/en-fr/products/reagents/flow-cytometry-reagents/research-reagents/single-color-antibodies-ruo/pe-rat-anti-mouse-cd21-cd35.552957
• anti-mouse CD23 PECy7, B3B4, Sony Biotechnology, 1108065, N/A, https://www.sonybiotechnology.com/us/pe-cy7-anti-mouse-cd23-6
• anti-mouse CD93 APC, AA4.1, eBioscience, 17-5892-82, AB_469466, https://www.thermofisher.com/antibody/product/CD93-AA4-1-Antibody-clone-AA4-1-Monoclonal/17-5892-82
• anti-mouse CD43 BV510, S7, BD Pharmingen, 563206, AB_2738069, https://www.bdbiosciences.com/en-fr/products/reagents/flow-cytometry-reagents/research-reagents/single-color-antibodies-ruo/bv510-rat-anti-mouse-cd43.563206
• anti-mouse CD25 PE, PC61, BD Pharmingen, 561065, AB_10563211, https://www.bdbiosciences.com/en-fr/products/reagents/flow-cytometry-reagents/research-reagents/single-color-antibodies-ruo/pe-rat-anti-mouse-cd25.561065
• anti-mouse CXCR4 PE/Dazzle 594, L276F12, BioLegend, 146514, AB_2563683, https://www.biolegend.com/en-us/products/pe-dazzle-594-anti-mouse-cd184-cxcr4-antibody-9859
• anti-mouse CD86 PE-Cy5, GL1, eBioscience, 15-0862-82, AB_468778, https://www.thermofisher.com/antibody/product/CD86-B7-2-Antibody-clone-GL1-Monoclonal/15-0862-82
• anti-mouse TACI PE, eBio8F10-3, eBioscience, 12-5942-81, AB_837121, https://www.thermofisher.com/antibody/product/CD267-TACI-Antibody-clone-ebio8F10-3-Monoclonal/12-5942-81

• anti-mouse CD138 PE-Cy7, 281-2, BioLegend, 142514, AB_2562198, https://www.biolegend.com/en-us/products/pe-cyanine7-anti-mouse-cd138-syndecan-1-antibody-8601
• anti-mouse CD19 PerCP-Cy5-5, 1D3, BD Pharmingen, 551001, AB_394004, https://www.bdbiosciences.com/en-fr/products/reagents/flow-cytometry-reagents/research-reagents/single-color-antibodies-ruo/percp-cy-5-5-rat-anti-mouse-cd19.551001
• anti-human CD3 APC-H7, SK7, BD Pharmingen, 560176, AB_1645475, https://www.bdbiosciences.com/en-fr/products/reagents/flow-cytometry-reagents/research-reagents/single-color-antibodies-ruo/apc-h7-mouse-anti-human-cd3.560176
• anti-human CD14 APC-H7, MφP9, BD Pharmingen, 560180, AB_1645464, https://www.bdbiosciences.com/en-fr/products/reagents/flow-cytometry-reagents/research-reagents/single-color-antibodies-ruo/apc-h7-mouse-anti-human-cd14.560180
• anti-human CD16 APC-H7, 3G8, BD Pharmingen, 560715, AB_1727432, https://www.bdbiosciences.com/en-fr/products/reagents/flow-cytometry-reagents/research-reagents/single-color-antibodies-ruo/apc-h7-mouse-anti-human-cd16.560715
• anti-human CD19 V500, HIB19, BD Pharmingen, 561121, AB_10562391, https://www.bdbiosciences.com/en-fr/products/reagents/flow-cytometry-reagents/research-reagents/single-color-antibodies-ruo/v500-mouse-anti-human-cd19.561121
• anti-human CD38 PerCPCy5-5, HIT2, Sony Biotechnology, 2117610, N/A, https://www.sonybiotechnology.com/eu/percp-cy5-5-anti-human-cd38-13
• anti-human CD24 PE, ML5, BioLegend, 311106, AB_314855, https://www.biolegend.com/en-us/products/pe-anti-human-cd24-antibody-1805
• anti-human CD27 APC, M-T271, BD Pharmingen, 561400, AB_10645790, https://www.bdbiosciences.com/en-fr/products/reagents/flow-cytometry-reagents/research-reagents/single-color-antibodies-ruo/apc-mouse-anti-human-cd27.561400
• anti-human IgD PE-CF594, IA6-2, BD Pharmingen, 562540, AB_11153129, https://www.bdbiosciences.com/en-fr/products/reagents/flow-cytometry-reagents/research-reagents/single-color-antibodies-ruo/pe-cf594-mouse-anti-human-igd.562540
• anti-human IgM BV605, MHM-88, Sony Biotechnology, 2172615, N/A, https://www.sonybiotechnology.com/eu/brilliant-violet-605-trade-anti-human-igm-6
• anti-mouse CD8a AF700, 53-6.7, eBioscience, 56-0081-82, AB_494005, https://www.thermofisher.com/antibody/product/CD8a-Antibody-clone-53-6-7-Monoclonal/56-0081-82
• anti-mouse CD8b.2 FITC, 53-5.8, BioLegend, 140404, AB_10643587, https://www.biolegend.com/en-us/products/fitc-anti-mouse-cd8b-2-antibody-6764
• anti-mouse CD3e PE, 145-2C11, BD Pharmingen, 553063, AB_394596, https://www.bdbiosciences.com/en-fr/products/reagents/flow-cytometry-reagents/research-reagents/single-color-antibodies-ruo/pe-hamster-anti-mouse-cd3e.553063
• PNA Biotin, B-1075, Vector Laboratories, B-1075-5, N/A, https://vectorlabs.com/products/glycobiology/biotinylated-peanut-agglutinin-pna
• anti-mouse CCR6 BV785, 29-2L17, BioLegend, 129823, AB_2715923, https://www.biolegend.com/en-us/products/brilliant-violet-785-anti-mouse-cd196-ccr6-antibody-14749
• anti-mouse CD98 PE, RL388 , BioLegend, 128207, AB_1186107, https://www.biolegend.com/en-us/products/pe-anti-mouse-cd98-4f2-antibody-4922
• anti-mouse pS6 AF488, D57.2.2E, Cell Signaling Technology, #4803, AB_916158, https://www.cellsignal.com/products/antibody-conjugates/phospho-s6-ribosomal-protein-ser235-236-d57-2-2e-xp-rabbit-mab-alexa-fluor-488-conjugate/4803?site-search-type=Products&N=4294956287&Ntt=%234803&fromPage=plp&_requestid=868130
• Rabbit IgG isotype control AF488 , DA1E , Cell Signaling Technology, 2975, AB_10699151, https://www.cellsignal.com/products/antibody-conjugates/rabbit-da1e-mab-igg-xp-isotype-control-alexa-fluor-488-conjugate/2975?site-search-type=Products&N=4294956287&Ntt=rabbit+anti-mouse+af488+%09da1e+&fromPage=plp
• anti-activated Caspase 3 AF647, C92-605, BD Pharmingen, 560626, AB_1727414, https://www.bdbiosciences.com/en-ca/products/reagents/flow-cytometry-reagents/research-reagents/single-color-antibodies-ruo/alexa-fluor-647-rabbit-anti-active-caspase-3.560626
• Rabbit anti-mouse ATF4 , D4B8, Cell Signaling Technology, 11815, AB_2616025, https://www.cellsignal.com/products/primary-antibodies/atf-4-d4b8-rabbit-mab/11815. Flow cytometry described in Tellier et al., Nat Immunol., 2016 17(3):323-30.
• anti-rabbit IgG (H+L), F(ab')2 AF647, Goat, polyclonal, Cell Signaling Technology, 4414, AB_10693544, https://www.cellsignal.com/products/secondary-antibodies/anti-rabbit-igg-h-l-f-ab-2-fragment-alexa-fluor-647-conjugate/4414
• Streptavidin PE-Cy7, Sony Biotechnology, 2626030, https://www.sonybiotechnology.com/eu/pe-cy7-streptavidin-3
• 7-AAD, BioLegend, 420403, https://www.biolegend.com/de-at/productstab/7-aad-viability-staining-solution-1649
• Sytox Blue, Invitrogen, S34857, https://www.thermofisher.com/order/catalog/product/S34857?SID=srch-srp-S34857
• Live Dead Aqua, Invitrogen, L34965, https://www.thermofisher.com/order/catalog/product/L34965?SID=srch-srp-L34965
• Live Dead Blue, Invitrogen, L23105, https://www.thermofisher.com/order/catalog/product/L23105?SID=srch-srp-L23105
• CaspGLOW, Invitrogen, 88-7003-42, https://www.thermofisher.com/document-connect/document-connect.html?url=https%3A%2F%2Fassets.thermofisher.com%2FTFS-Assets%2FLSG%2Fmanuals%2F88-7003.pdf&title=VGVjaG5pY2FsIERhdGEgU2hlZXQ6IENhc3BHTE9XJnRyYWRlOyBGbHVvcmVzY2VpbiBBY3RpdmUgQ2FzcGFzZSBTdGFpbmluZyBLaXQ=
• BODIPY-C11, Invitrogen, D3861, https://www.thermofisher.com/order/catalog/product/fr/fr/D3861
• Depleting anti-mouse CD8a, 2.43, BioXCell, BE0061, https://bioxcell.com/invivomab-anti-mouse-cd8a-be0061
• Depleting Rat IgG2b control Isotype, LTF-2, BioXCell, BE0090, https://bioxcell.com/invivomab-rat-igg2b-isotype-control-anti-keyhole-limpet-hemocyanin-be0090
• anti-HELLS, Rabbit, polyclonal, ProteinTech , 11955-1-AP, AB_2117529, https://www.ptglab.com/fr/products/HELLS-Antibody-11955-1-AP.htm
• Mouse monoclonal anti-α Tubulin, DM1A, Sigma, T6199, AB_2617116, https://www.sigmaaldrich.com/FR/fr/search/t6199?focus=products&page=1&perpage=30&sort=relevance&term=t6199&type=product
• IRDye® 680RD Goat anti-Mouse IgG, Goat, polyclonal, LI-COR Biosciences, 926-68070, AB_10956588, https://www.licor.com/bio/reagents/irdye-680rd-goat-anti-mouse-igg-secondary-antibody
• IRDye® 800CW Goat anti-Rabbit, Goat, polyclonal, LI-COR Biosciences, 926-32211, AB_621843, https://www.licor.com/bio/reagents/irdye-800cw-goat-anti-rabbit-igg-secondary-antibody
• Goat anti-mouse Ig, Goat, polyclonal, SouthernBiotech, 1010-01, AB_2794121, https://www.southernbiotech.com/goat-anti-mouse-ig-human-ads-unlb-1010-01
• HRP-Goat anti-mouse IgM, Goat, polyclonal, SouthernBiotech, 5300-05B, AB_2794201, https://www.southernbiotech.com/sba-clonotyping-system-c57bl-6-hrp-5300-05b
• HRP-Goat anti-mouse IgG1, Goat, polyclonal, SouthernBiotech, 5300-05B, AB_2794201, https://www.southernbiotech.com/sba-clonotyping-system-c57bl-6-hrp-5300-05b
• HRP-Goat anti-mouse IgG2b, Goat, polyclonal, SouthernBiotech, 5300-05B, AB_2794201, https://www.southernbiotech.com/sba-clonotyping-system-c57bl-6-hrp-5300-05b

• HRP-Goat anti-mouse IgG2c, Goat, polyclonal, SouthernBiotech, 5300-05B, AB_2794201, https://www.southernbiotech.com/sba-clonotyping-system-c57bl-6-hrp-5300-05b
• HRP-Goat anti-mouse IgG3, Goat, polyclonal, SouthernBiotech, 5300-05B, AB_2794201, https://www.southernbiotech.com/sba-clonotyping-system-c57bl-6-hrp-5300-05b
• HRP-Goat anti-mouse IgA, Goat, polyclonal, SouthernBiotech, 5300-05B, AB_2794201, https://www.southernbiotech.com/sba-clonotyping-system-c57bl-6-hrp-5300-05b
• Purified mouse IgM, SouthernBiotech, 5300-01B, AB_2796077, https://www.southernbiotech.com/c57bl-6-mouse-immunoglobulin-panel-5300-01b
• Purified mouse IgG1, SouthernBiotech, 5300-01B, AB_2796077, https://www.southernbiotech.com/c57bl-6-mouse-immunoglobulin-panel-5300-01b
• Purified mouse IgG2b, SouthernBiotech, 5300-01B, AB_2796077, https://www.southernbiotech.com/c57bl-6-mouse-immunoglobulin-panel-5300-01b
• Purified mouse IgG2c, SouthernBiotech, 5300-01B, AB_2796077, https://www.southernbiotech.com/c57bl-6-mouse-immunoglobulin-panel-5300-01b
• Purified mouse IgG3, SouthernBiotech, 5300-01B, AB_2796077, https://www.southernbiotech.com/c57bl-6-mouse-immunoglobulin-panel-5300-01b
• Purified mouse IgA, SouthernBiotech, 5300-01B, AB_2796077, https://www.southernbiotech.com/c57bl-6-mouse-immunoglobulin-panel-5300-01b

Eukaryotic cell lines

Policy information about cell lines and Sex and Gender in Research

Cell line source(s)

293T : ATCC CRL-3216
40LB feeder cell line was provided by D. Kitamura (Research Institute for Biomedical Sciences, Tokyo University, Japan)

Authentication

None of the cell lines were authenticated.

Mycoplasma contamination

Cell lines tested negative for mycoplasma contamination as monitered by HEK-Blue Mycoplasma detection kit (Invivogen).

Commonly misidentified lines
(See ICLAC register)

None

Animals and other research organisms

Policy information about studies involving animals; ARRIVE guidelines recommended for reporting animal research, and Sex and Gender in Research

Laboratory animals

Mice with with LoxP sites flanking Hells exons 9 and 10 (HellsF allele43) were crossed with CD21-Cre (Tg(Cr2-cre)3Cgn) (or Mb1-Cre (Cd79atm1(cre)Reth strains. CD21-Cre and Mb1-Cre mice were kindly provided by Patrick Revy (Institut Imagine, Paris, France) and Simon Fillatreau (INEM, Paris, France), respectively. The resulting offspring were inter-crossed and maintained on a mixed C57BL/6J:129 genetic background at the SFR Necker animal facility (Paris), in individual ventilated cages with enrichment, under specific and opportunistic pathogen-free (SOPF) conditions. Animals were fed standard chow diet (Teklad Global 2918, 18% protein, irradiated) ad libitum, and kept under ambient temperature (21–22°C) and 50–60% humidity, with 12h-12h on-off light cycle. 6 weeks-old to 6 months-old mice of both sex were used for all experiments. For the treatment with DNMT1 inhibitor, 6 week-old male and female C57BL/6J SOPF mice were purchased from Charles River (France).

Wild animals

No wild animals were used for this study

Reporting on sex

No sex difference were reported in this study. Groups were equally mixed.

Field-collected samples

This study does not involve field-collected samples.

Ethics oversight

The research described in this article complies with all relevant ethical regulations. All animal studies were ethically reviewed by Ethics Commitee of Paris Descartes University (CEEA34, authorizations n° APAFiS #6691-2016062816418985, and APAFiS #31365-2021030513547842) and Anses/EnvA/Upec Ethics Committee (C2EA-16, authorization n°APAFiS #9951-2017051611079789), validated by the French Ministry of Research, and carried out in accordance with the European Directive 2010/63/EEC.

Note that full information on the approval of the study protocol must also be provided in the manuscript.

Flow Cytometry

Plots

Confirm that:

☒ The axis labels state the marker and fluorochrome used (e.g. CD4-FITC).

☒ The axis scales are clearly visible. Include numbers along axes only for bottom left plot of group (a 'group' is an analysis of identical markers).

☒ All plots are contour plots with outliers or pseudocolor plots.

☒ A numerical value for number of cells or percentage (with statistics) is provided.

Methodology

Sample preparation	Single cell suspensions were prepared from mouse spleen, bone marrow, and Peyer's Patches in cold HBSS medium with Ca2 + and Mg2+ (Gibco) supplemented with 10% FBS (Fetal Bovine Serum, Gibco), and filtered through a 40 µm nylon mesh (BD Biosciences). Red blood cells from spleen and bone marrow were lysed by hypotonic shock thanks to a 30 s incubation at room temperature (RT) in erylysis buffer (0.747% NH4Cl, 1.7 mM Tris-HCl pH 7.2), followed by immediate addition of cold HBSS-10% FBS and centrifugation at 300 g for 5 min at 4°C. Cells were washed in HBSS-10% FBS and counted automatically on Cellometer (Nexcelom Bioscience) after exclusion of dead cells with trypan blue. Surface staining of unfixed cells with fluorochrome-conjugated antibodies were performed on 1-10 M cells in 100 µl of PBA (PBS without Ca2+Mg2+ 491 (Gibco), supplemented with 0.5 % BSA) for 20 min on ice in the dark, except for iGB cell cultures that were stained at RT. Cells were subsequently washed with PBA and finally resuspended in cold PBS-2% FBS-2 mM EDTA.
Instrument	BD FACS Canto II, BD LSRFortessa, BD FACS Aria III , BD FACS Aria II SORP.
Software	Flow cytometry data was acquired using BD Diva Software. Data analysis was performed using FlowJo (Treestar) software v10.8.1
Cell population abundance	B Cell purification from naive mice was performed either on BD FACS Aria II SORP or III, or using EasySepTM Mouse B Cell Isolation Kit (Stemcell Technologies).<br>Germinal Center B cell purification : Performed on BD FACS Aria II SORP or III. In control cohorts, around 3 to 5% of total B220 + cells (from 100.000 to 300.000 purified cells from one spleen), and in Hells deficient mice ranging from 0.3 to 1.5% of total B220+ cells in the spleen (15.000 to 50.000 cells from one spleen). Sorting for purification was performed twice, on the same gating strategy with increased purification parameters on the sorter to allow for a greater than 95% purity.<br>Memory B cells purification : Performed on BD FACS Aria II SORP or III. Sorting for purification was performed twice, after enrichment on IgG1 parameter.
Gating strategy	For Flow cytometry, all cell populations were gated on live singlets (7-AAD/LiveDeadAqua/LiveDeadBlue/SytoxBlue negative) and consecutively gated on B220+ populations (Extended Data Figure 9). Further gating strategy for each populations is detailed in figures.

☒ Tick this box to confirm that a figure exemplifying the gating strategy is provided in the Supplementary Information.
